# Membrane fusogenic nanoparticle‐based HLA‐peptide‐addressing universal T cell receptor‐engineered T (HAUL TCR‐T) cell therapy in solid tumor

**DOI:** 10.1002/btm2.10585

**Published:** 2023-08-07

**Authors:** Ruihan Xu, Qin Wang, Junmeng Zhu, Yuncheng Bei, Yanhong Chu, Zhichen Sun, Shiyao Du, Shujuan Zhou, Naiqing Ding, Fanyan Meng, Baorui Liu

**Affiliations:** ^1^ The Comprehensive Cancer Centre of Nanjing Drum Tower Hospital The Affiliated Hospital of Nanjing University Medical School Nanjing China

**Keywords:** HLA‐peptide complex, membrane fusogenic nanoparticles, solid tumors, TCR‐T cell therapy, tumor cell membrane modification

## Abstract

T cell receptor‐engineered T (TCR‐T) cell therapy has demonstrated therapeutic effects in basic research and clinical trials for treating solid tumors. Due to the peptide‐dependent recognition and the human leukocyte antigen (HLA)‐restriction, TCR‐T cell therapy is generally custom designed to target individual antigens. The lack of suitable universal targets for tumor cells significantly limits its clinical applications. Establishing a universal TCR‐T treatment strategy is of great significance. This study designed and evaluated the HLA‐peptide‐addressing universal (HAUL) TCR‐T cell therapy based on HLA‐peptide (pHLA) loaded membrance fusogenic deliver system. The pHLA‐NP‐based tumor cell membrane modification technology can transfer the pHLA onto the surface of tumor cells through membrane fusogenic nanoparticles. Then tumor cells are recognized and killed by TCR‐T cells specifically. The HAUL TCR‐T cell therapy technology is a universal technology that enables tumor cells to be identified and killed by specific TCR‐T cells, regardless of the HLA typing of tumor cells.

**Translational Impact Statement**

We presented for the first time the addition of peptide‐human leukocyte antigen on the tumor cell membrane as the target for T cell receptor‐engineered T (TCR‐T) cell‐specific recognition without HLA restriction, which solved the problem of the lack of effective targets for TCR‐T cell therapy. This article proposed a proof of principle for the HAUL TCR‐T cell therapy, which had translational potential in clinical application for solid tumors.

## 1 INTRODUCTION

T cell receptor‐engineered T (TCR‐T) cells recognize the peptide‐human leukocyte antigen (pHLA) on tumor cells through transfected TCR molecules. Peptides presented by human leukocyte antigen (HLA) derive from proteins of any subcellular location, broadening the target spectrum of tumor immunotherapy. TCR is an entirely anthropogenic structure, unlikely to cause immune rejection. Compared with CAR‐T, TCR‐T is easier to penetrate solid tumors.^1^

In recent years, nanomaterials have been widely used for the delivery of antigens or drugs or activated immune cells selectively to target sites, thus decreasing their toxicity and improving efficacy.^2^, ^3^ The current strategy of antigens delivery is to load peptides onto HLA molecules on the surface of tumor cells. For example, injecting a specific antigen into the tumor,^4^ using a carrier such as polymer nanospheres to systematically deliver the antigen to the local tumor for specific release^5^, ^6^ can cause an immune response against the antigen, making the antigen not initially expressed in the tumor cells can be effectively recognized and killed by specific T cells. Ji‐Ho Park et al. reported that optimized and improved membrane fusogenic liposome (MFL) was tumor‐specific and widely labeled in tumor tissues^7^ which can load artificial targets on tumor tissues to support subsequent targeted therapy.^8^ A collaborative targeting system using MFL as delivery media may be a powerful method for loading TCR‐T targets.

TCR‐T cell therapy has shown specific anti‐tumor effects in basic research and clinical trials for treating solid tumors. However, the lack of suitable pHLA targets on tumor cells severely restricts the clinical application of TCR‐T cell therapy. First, the activation of TCR‐T depends on HLA molecules presenting tumor antigens. HLA typing mismatch limits TCR‐T activation. Second, Tumor heterogeneity is one of the major problems in treating solid tumors. For example, the expression of NY‐ESO‐1 is only about 10% in gastric cancers.^9^, ^10^ Coupled with the limitation of HLA, only a small number of patients can consider the existing NY‐ESO‐1 TCR‐T cell therapy. Lastly, some tumors have immune escape phenomena such as HLA down‐regulation and loss of neoantigens, resulting in the lack of T cell recognition targets on the surface of tumor cells.^11^, ^12^ Therefore, TCR‐T cell therapy is challenging to have universal application value. It is much‐needed for TCR‐T cell therapy with broader coverage and more powerful functions.

We established an HLA‐peptide‐addressing universal T cell receptor‐engineered T (HAUL TCR‐T) cell therapy treatment strategy. In the study, we modified pHLA to the surface of tumor cells based on membrane fusogenic nanoparticles (NPs). We used the HLA‐A2‐restricted NY‐ESO‐1 antigen peptide (NY‐ESO‐1_157‐165_) as the model antigen, prepared its pHLA molecule in vitro, then loaded the pHLA onto the tumor cell membrane with the prepared NPs. We evaluated the anti‐tumor effect of this HAUL TCR‐T technology in vitro and in vivo. This method facilitates the implementation of TCR‐T treatment and has excellent potential in treating solid tumors.

## 2 MATERIALS AND METHODS

### 2.1 Cell lines

Human gastric cell (GC) line MKN45, ffLuc+MKN45, NUGC4, SNU719, HGC27, and human gastric epithelial cell line GES‐1 were cultured in RPMI 1640 medium supplemented with 10% fetal bovine serum (FBS), 100 U/mL penicillin and 100 μg/mL streptomycin. NIH3T3 and human embryonic kidney 293T (HEK‐293T) were cultured in DMEM medium supplemented with 10% fetal calf serum, 100 IU/mL penicillin, and 100 μg/mL streptomycin. All cells were incubated at 37°C and 5% CO_2_, authenticated by checking morphology by microscopy after plating at different concentrations. Only Mycoplasma‐free cells were used.

### 2.2 Preparation of membrane fusogenic nanoparticles

For membrane fusogenic NPs preparation, 1,2‐Dimyristoyl‐sn‐glycero‐3‐phosphocholine (DMPC; Avanti Polar Lipids, USA), 1,2‐distearoyl‐sn‐glycero‐3‐phosphoethanolamine‐N‐ [maleimide (polyethylene glycol)‐3400] (DSPE‐PEG3400‐MAL; Laysan Bio, USA) and 1,2‐dioleoyl‐3‐trimethylammonium‐propane (DOTAP; Cordenpharma, Switzerland) were used to prepare NPs. The molar ratios of DMPC, DSPE‐PEG3400‐MAL, and DOTAP were 1:1:1 (the mass ratio, 2.95: 1: 0.78). Lipids were dissolved in chloroform and then completely dried. The remaining lipid film was hydrated using phosphate‐buffered saline (PBS) and then extruded through 100 nm membrane pores (Whatman, UK). The hydrodynamic size and zeta potential of NPs were measured using dynamic light scattering (DLS) (Zetasizer Nano‐ZSE; Malvern, UK). Quality control was also done using transmission electron microscopy (TEM).

### 2.3 Preparation of pHLA‐NP

Recombinant pHLA class I was produced by expressing glycine‐serine (GS) linker‐tethered single‐chain pHLA class I complexes in bacteria, followed by dilution refolding and purification via size exclusion chromatography (SEC) or Nickel column. The peptide was tethered to the amino‐terminal end of the light chain via a flexible GS linker, and the heavy chain was engineered to encode a 6xHis tag and a free Cysteine at their carboxy‐terminal end. pHLAs were coated onto NPs. pHLAs carrying a free carboxyterminal Cysteine were conjugated to the maleimide‐functionalized NPs in PBS overnight at room temperature. The pHLA‐conjugated NPs (pHLA‐NP) were sterilized by filtration through 0.22 μm filters and stored in PBS at 4°C. pHLA content was measured using Bradford assay (Bio‐Rad Laboratories, USA) and SDS‐PAGE.

### 2.4 Generation of NY‐ESO‐1 TCR‐T cells

The blood collection procedure was carried out following the guidelines verified and approved by the Ethics Committee of Drum Tower Hospital. PBMCs were isolated by centrifugation on a Ficoll density gradient and suspended in AIM‐V media (Gibco, USA) supplemented with 10% FBS. Cells were frozen in 90% FBS serum (Gibico, USA) and 10% dimethyl sulfoxide (Sigma, USA). All PBMCs were either used for experiments or stored in a secure liquid nitrogen freezer until use.

PBMCs were cultured by adherence for 2–3 h, and non‐adherent cells were moved and suspended in an AIM‐V medium supplemented with 50 ng/mL OKT‐3 (eBioscience, USA) and 300 U/mL human recombinant IL‐2 (eBioscience, USA) for 2–3 days. Cells were transfected with the PB transposon NY‐ESO‐1 TCR plasmid and PB transposase plasmid by Nucleofector 2B (Lonza, Germany) using the Amaxa Human T cells Nucleofector Kit, VPA‐1002 (Lonza, Germany). 1 × 10^7^ cells were washed twice with 37°C pre‐warmed PBS by centrifuging at 800 rpm for 5 min and resuspended in 100 μL transfection buffer and then transferred into the electroporation cuvette. After electroporation, cells were resuspended in 4 mL 37°C pre‐warmed AIM‐V medium containing 10% FBS, moved into a 12‐well cell plate, and incubated at 37°C in 5% CO_2_. Cells were transferred into a fresh complete AIM‐V medium containing IL‐2 (100 U/mL), IL‐7 (10 ng/mL), and IL‐15 (10 ng/ mL) 4 h after electroporation, and the cells culture medium was half replaced every 2–3 days. The table transformation efficiency was evaluated by tetramer detection 4 days after electroporation.

### 2.5 Flow cytometry

Antigen‐specific T cells and tumor cells were harvested and stained with mouse anti‐human antibodies labeled with fluorescent markers for 40 min at 4°C in the dark as follows: CD8‐FITC (RPA‐T8, BD Biosciences), NY‐ESO‐1 Tetramer‐SLLMWITQC‐APC (MBL Beijing Biotech), HLA‐02‐PE (BB7.2, BD Biosciences), HLA‐02‐APC (BB7.2, eBioscience), His‐tag iFluor 488 (Genscript), the T cell activation marker 4‐1BB (CD137) (BD Biosciences). The Cytometric Bead Array (CBA) human IFN‐γ flex set (BD Biosciences) was used for cytokine detection. FACS CaliburTM and BD AccuriTM C6 were used to perform fluorescent expression analysis.

### 2.6 Confocal imaging

We observed through a confocal microscope whether pHLA‐NP could transfer the pHLA onto the surface of tumor cell membranes. We used His‐tag to locate the pHLA monomer, Dil to mark the cell membrane, and DAPI to stain the nucleus. We took NUGC4, MKN45, and HGC27 as target cells. When the cells grew to the logarithmic proliferation stage, they were planted in a 4‐Chamber Glass Bottom Dish (Cellvis). After the cells adhered to the wall, discarded the culture medium, added pHLA‐NP, and incubated at 37°C, 5% CO_2_ for 1 h. After fixing with 4% paraformaldehyde and blocking with immunostaining blocking solution, added His‐tag iFluor 488, DiI, and incubated at room temperature for 40–50 min; Finally, stained the nucleus with DAPI, incubated at room temperature for 3–5 min, and added anti‐Fluorescence quenching mounting fluid. We observed whether the pHLA was located on the cell membrane under a laser confocal microscope. And took SUN719, GES‐1, HEK‐293T, and NIH3T3 cells to test the tumor targeting of pHLA‐NP.

### 2.7 Cytotoxicity assay

Transduced T cells were tested for lytic activities by Carboxyfluorescein succinimidyl ester (CFSE)/Propidium iodide (PI) labeling cytotoxicity assay. Target tumor cells were labeled with 4 μM CFSE (Invitrogen) for 10 min at 37°C in PBS. Labeling was stopped by adding 10‐fold volume and extensively washed in PBS before seeding into the flow tubes. CFSE‐labeled cells were treated with pHLA‐NP or NP for 1 h and then incubated with TCR‐T cells by different effector‐to‐target (E: T) ratios for 6 h. PI (Sigma) was added to determine the percentage of cell death. Samples were analyzed by flow cytometry.

### 2.8 In vivo anti‐tumor efficacy

The Ethics Committee of Nanjing Drum Tower Hospital approved all experiments in this study. All mice were kept in the specific pathogen‐free (SPF) Laboratory Animal Center of Affiliated Nanjing Drum Tower Hospital of Nanjing University Medical School. Randomized number method was used for grouping. Data statisticians do not know the grouping.

For the subcutaneous tumor model, 4–5‐week‐old male BALB/c nude mice were injected with ffLuc+MKN45 cells (3 × 10^6^ suspended cells in 100 μL PBS). The mice were divided into four groups: NS group, pHLA‐NP group, NY‐ESO‐1 TCR‐T group, and pHLA‐NP + NY‐ESO‐1 TCR‐T group (HAUL TCR‐T group), every group had five mice. At the adoptive T cell transfer, mice were weighed and inspected for tumor development. The weights of the mice were recorded every 3 days. The tumor size was determined using a caliper. Assuming the tumor shape was an ellipsoid, the volume was calculated as length × width^2^ × 0.5. The results of computed tomography (CT) and bioluminescence were taken at the observation endpoint. The situation of subcutaneous tumor nodules was assessed by luciferase‐mediated bioluminescence imaging (BLI) technology: NS group (*n* = 5), pHLA‐NP group (*n* = 6), NY‐ESO‐1 TCR‐T group (*n* = 6), and HAUL TCR‐T group (*n* = 7).

For the peritoneal metastasis tumor model, 4–5‐week‐old male BALB/c nude mice were injected intraperitoneally with firefly luciferase MKN45 cells expressing firefly luciferase stably (ffLuc^+^ MKN45 cells) (3 × 10^6^ suspended cells in 200 μL NS). On the 7th day after the tumor was implanted, the mice were observed by BLI. The mice were randomly divided into four groups. In the tumor suppression experiments, every group had 4 mice. In the survival experiment, every group had 5 mice. Treatment was performed on the 10th and 15th days after the tumor was implanted. The intraperitoneal injection of pHLA‐NP 100 ug per and NY‐ESO‐1 TCR‐T cells 1 × 10^7^ per time at an interval of 4 h. And on the 21st and 28th days, BLI was performed to observe the tumor growth in the abdominal cavity. The weights of the mice were recorded twice a week. Three mice were randomly selected from each group to test liver and kidney function.

One mouse was randomly selected from each group, and the main organs were collected for histology analysis. Organs were fixed in 4% paraformaldehyde, embedded in paraffin, sliced, and stained with hematoxylin–eosin (H&E).

### 2.9 Statistical analysis

Graph**P**ad Prism 7.0a was used for all statistical analyses. The variance was similar between the groups that were compared statistically. No statistical methods were used to predetermine the sample size. Data were expressed as Mean ± SEM in the individual experiments, and the differences between the groups were determined using a two‐tailed Student's *t*‐test. *p* < 0.05 was considered statistically significant, as indicated with asterisks (*p < 0.05, **p < 0.01, ***p < 0.001).

## 3 RESULTS

### 3.1 Generation and characterization of pHLA‐NP

**FIGURE 1:**
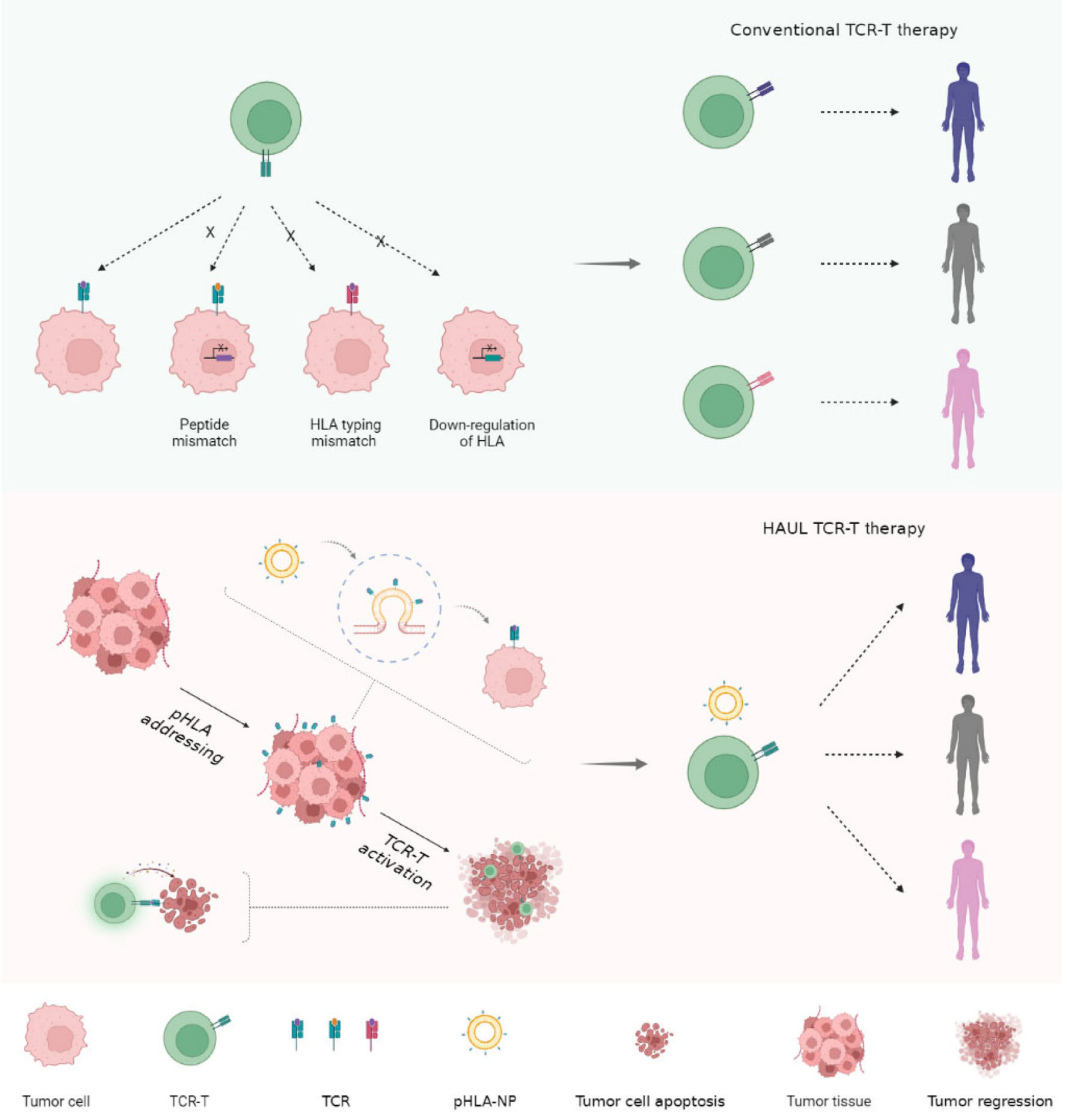
Schematic diagram of the HAUL TCR‐T cell therapy. TCR‐T cells recognize the pHLA on tumor cells' surfaces through antigen‐specific TCR transfected by T cells, which must match HLA typing and peptide. Conventional TCR‐T cell therapy is generally custom designed to target individual antigens. We created the HAUL TCR‐T cell therapy technology enables tumor cells to be identified and killed by specific TCR‐T cells, regardless of the HLA typing of tumor cells.

**FIGURE 2:**
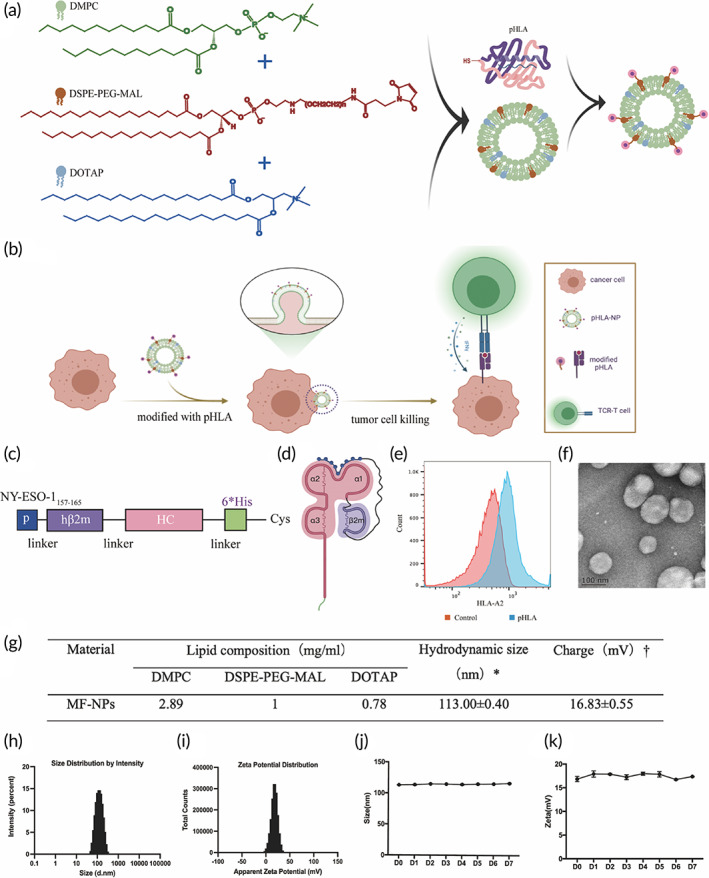
The construction of pHLA‐NPs and the proposed immunotherapeutic mechanism of HAUL‐TCR‐T therapy. (a) The schematic diagram of the synthesis of lipid‐conjugated pHLA‐NPs. (b) The schematic illustration demonstrates pHLA‐NPs modified pHLA onto tumor cell membranes via membrane fusion. pHLA modification of tumor cell membrane provides specific targets for TCR‐T cell recognition, enabling TCR‐T cell to exert antitumor effects independent of tumor inherent target profiles. (c) Primary structure of single‐chain pHLA class I complexes, using flexible GS linkers to tether a 9mer peptide (NY‐ESO‐1_157‐165_) to β2m, the heavy chain and a carboxyterminal tail, a Hisx6 tag, and a free Cysteine. (d) Cartoons depicting the folded trimolecular pHLA class I. (e) The HLA‐A2 fluorescence intensity of the microspheres connected with (pHLA group) or without the pHLA (control group). (f) Transmission electron microscope image of NPs. (g) Preparation and characterization of NPs. Scale bar, 100 nm. (h) DLS to detect the particle size of NPs. (i) DLS to detect the charge of NPs. (j) DLS to detect NPs within 1 week change in size. (k) DLS detects the charge of NP within 1 week.

TCR‐T cells recognize the pHLA on tumor cells through transfected antigen‐specific TCR. HLA mismatching and down‐regulation/loss of HLA and antigens resulted in the lack of T cell recognition targets on the surface of tumor cells. TCR‐T cell therapy is generally custom designed to target individual antigens at present. We invented the HAUL TCR‐T cell therapy technology that enables tumor cells to be identified and killed by specific TCR‐T cells, regardless of the HLA typing of tumor cells (Figure 1). pHLA‐NPs modified pHLA onto tumor cell membranes via membrane fusion. pHLA modification of tumor cell membrane provides specific targets for TCR‐T cell recognition, enabling TCR‐T cell to exert antitumor effects independent of tumor inherent target profiles (Figure 2a,b).

The peptide was tethered to the amino‐terminal end of the β2‐microglobulin (β2m) via a flexible glycine‐serine (GS) linker. The heavy chain was engineered to encode a 6xHis tag and a free Cysteine at their carboxy‐terminal end (Figure 2c). The pHLA protein (Figure 2d) was successfully expressed in *Escherichia coli* BL21 (Figure S1a), with a molecular weight of approximately 49.4 kDa in SDS‐PAGE (Figure S1b), followed by dilution refolding and purification via a nickel‐nitrilotriacetic acid (Ni‐NTA) column (Figure S1c,d). The pHLA protein was coated on latex microspheres and detected by HLA‐A2 conformation‐specific monoclonal antibody W6/32 (PE) or HLA‐02‐PE. The fluorescence intensity of the microspheres connected with the pHLA increased, indicating that the pHLA were folded into the correct spatial conformation (Figure 2e). The TEM image showed that the NPs were approximately spherical and well dispersed (Figure 2f). The particle size measured by the TEM was basically the same as measured by DLS (Figure 2f,g). The average particle size of NPs measured by DLS was 113.00 ± 0.40 nm (Figure 2g,h), and the average charge was 16.83 ± 0.55 mV (Figure 2g–i). DLS monitored the changes in particle size and charge of NPs in PBS solution for a week. The size and charge fluctuated little, indicating that NPs were stable in the buffer (Figure 2j,k). The C‐terminal cysteine residue of pHLA provided the free sulfhydryl group to connect with the maleimide group of DSPE‐ PEG‐Mal through Michael addition reaction. (Figure S1e,g). pHLA‐NPs did not affect the viability of tumor cells (Figure S2a–c).

### 3.2 pHLA‐NP could transfer the pHLA onto the surface of tumor cells

**FIGURE 3:**
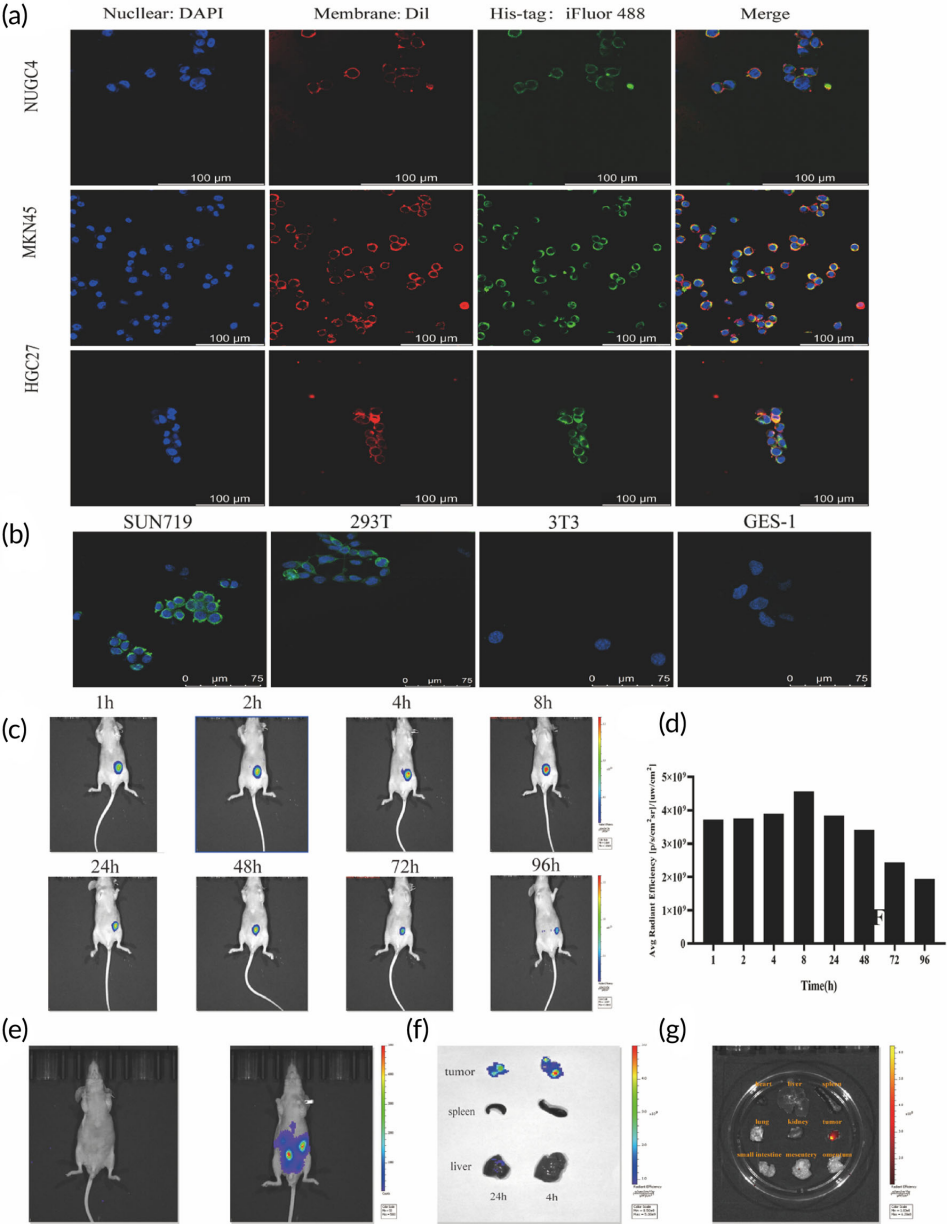
Tumor membrane targeting detection of pHLA‐NP. (a) Localization of pHLA modified by pHLA‐NP on tumor cells NUGC4, MKN45, and HGC27. Nucleus (DAPI, blue), cell membrane (DiI, red), and pHLA (His‐tag antibody, green). Scale bars, 100 μm. (b) Localization of pHLA modified by pHLA‐NP on tumor cell SUN719, non‐tumor cells 293T, 3T3, and GES‐1. Nucleus (DAPI, blue) and pHLA (His‐tag antibody, green). Scale bars, 75 μm. (c, d) The dynamics of Cy5 signal over time within tumor tissue after injection of Cy5‐labeled pHLA‐NP into the tumor. (e) In vivo bioluminescence images of peritoneal metastatic tumor‐bearing mice. (f) The Cy5 signals in the tumor, spleen, and liver on the 4th and 24th hours after intraperitoneal injection of Cy5‐labeled pHLA‐NP. (g) The Cy5 signals in the tumor and other organs at the 48th hour after intraperitoneal injection of Cy5‐labeled pHLA‐NP.

Next, we observed through a confocal microscope whether pHLA‐NP could transfer the pHLA to tumor cell membranes. Anti‐His‐tag antibody was used to detect the pHLA molecules. The cell membrane and nucleus were stained with Dil and DAPI. Confocal images indicated that pHLA was co‐localized with the cell membrane dye DiI, indicating that pHLA was successfully transferred to the tumor cell membrane (Figure 3a; Figure S3). Tumor cell line SNU719 was also modified with pHLA molecules. By comparison, confocal images indicated that none or a few pHLA could be transferred to the membranes of non‐tumor cells 293T, NIH3T3, and GES‐1 (Figure 3b). The density of cell surface pHLA was controllable. Theoretically, only 7.4 ug NPs were needed for a complete reaction with 100 ug pHLA. We next studied the dose‐response of NUGC4 binding of NPs. NPs up to 100 μg/mL still did not lead to 100% coating of 10^6^ NUGC4 cells, and pHLA‐NPs up to 1000 μg/mL still did not lead to 100% coating of 10^6^ NUGC4 cells (Figure S1f,h).

Then we evaluated the distribution of pHLA‐NPs in vivo by intratumoral and intraperitoneal injection. Cy5‐NHS was used to label the pHLA, and the pHLA‐NPs were injected intratumorally into tumor‐bearing nude mice. The photographs were taken with an infrared imager, and the fluorescence signals of the tumor site were quantified. It could be seen from the quantitative graph of the near‐infrared fluorescence signal at the tumor site that at the tumor site, there was no apparent fluorescence attenuation in the first 24 h, and there was still a certain fluorescence intensity after 96 h (Figure 3c,d). Cy5‐NHS was used to label pHLA, and pHLA‐NP was intraperitoneally injected into firefly luciferase (ffluc)‐tumor‐bearing nude mice (Figure 3e). The results showed that pHLA‐NP had reached the tumor site after 4 h. There was still a certain amount of fluorescence under the infrared imager at the 24th hour (Figure 3f). Take ex vivo imaging of the tumor and other organs at the 48th hour. Only the tumor has fluorescent expression (Figure 3g). The above results suggested that pHLA‐NPs modification facilitates cooperation with TCR‐T cells via different routes.

### 3.3 In vitro function verification of HAUL TCR‐T

**FIGURE 4:**
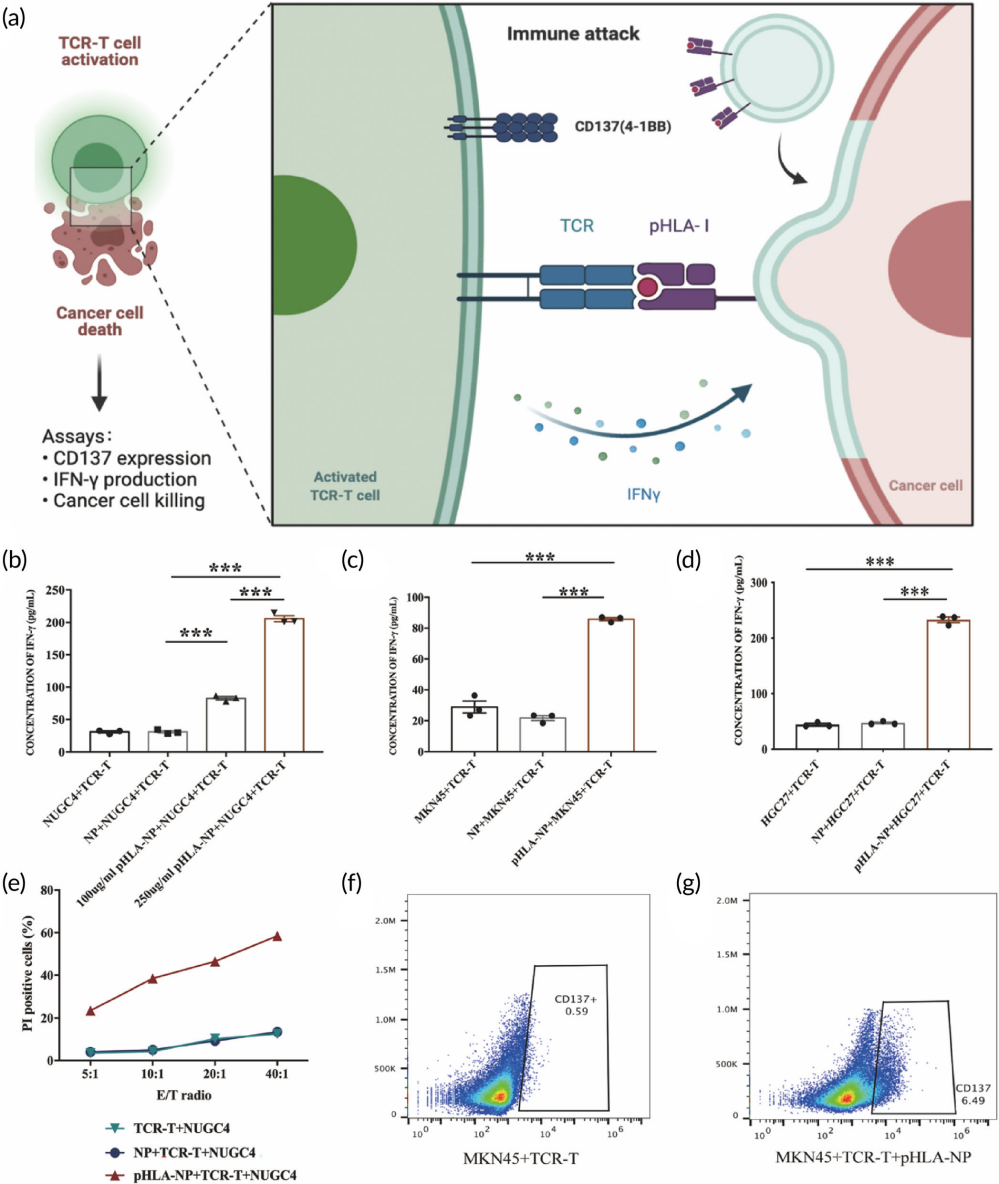
Functional verification of pHLA‐NP in vitro. (a) Schematic illustration of action mode of HAUL‐TCR‐T therapy and evaluation of in vitro antitumor response. pHLA‐NP and NPs were co‐cultured with different tumor cells for 1 h in advance and then washed with PBS 1–2 times as target cells. Assessment of IFN‐γ in co‐culture supernatants with (b) NUGC4, (c) MKN45, (d) HGC27 at E: T ratio of 10:1. (Data are represented as Mean ± SEM, *n* = 3. Student's *t*‐test was used for statistical analysis. ***p < 0.001.) (e) Cytotoxicity of TCR‐T cells toward target cells. CFSE‐labeled NUGC4 was co‐cultured with pHLA‐NP or NPs for 1 h as target cells. TCR‐T cells and target cells were co‐cultured at E: T of 5:1, 10:1, 20:1, and 40:1, respectively, PI was added 6 h after incubation, and the percentage of dead tumor cells was analyzed by flow cytometry. (f, g) Quantification of CD137 expression on NY‐ESO‐1 TCR‐T in co‐culture with MKN45.

Given that pHLA could be effectively modified on the tumor cell membrane by pHLA‐NPs, it was further investigated whether the modified pHLA could activate the corresponding TCR‐T cells (Figure 4a). Tumor cells were treated with pHLA‐NP or NPs, and cocultured with TCR‐T cells at an E: T ratio of 10:1. The proportion of NY‐ESO‐1‐specific TCR‐T cells detected by NY‐ESO‐1_157‐165_‐HLA‐A*02:01 tetramer was over 5% (Figure S4). The IFN‐γ level in the pHLA‐loaded NUGC4 group was higher than that of the unmodified NUGC4 control group. With the increase in pHLA concentration, the level of IFN‐γ also increased (*p* < 0.001). In addition, there was no significant difference in IFN‐γ secretion between the NUGC4 control group and the NP‐treated NUGC4 group. The increased IFN‐γ secreted by TCR‐T cells is due to the pHLA loaded in the tumor cell membrane can be recognized explicitly by TCR‐T cells (Figure 4b). Consistent with the above results, the IFN‐γ secretion levels in pHLA‐NP‐loaded HGC27 or MKN45 groups were significantly increased in contrast with HGC27 or MKN45 control groups (*p* < 0.001) (Figure 4c,d). CFSE‐labeled NUGC4 cells were modified with pHLA‐NP or NPs and cocultured with TCR‐T cells using an E: T ratio of 5:1, 10:1, 20:1, and 40:1, respectively. TCR‐T cells mediated potent T‐cell cytotoxicity in an E: T ratio‐dependent manner. When the E: T ratio was 40:1, the maximum killing ratio was 58.3%. The killing ratios of NUGC4 control and NPs‐treated NUGC4 groups were 12.5% and 13.5% under the same E: T ratio (Figure 4e). Killing rates of tumor cells were associated with the dose of nanoparticles (Figure S5). In addition, the expression of CD137, a T cell activation marker, increased significantly in the pHLA‐loaded MKN45 group. (Figure 4f,g).

### 3.4 HAUL TCR‐T inhibited the progression of the human GC subcutaneous transplantation model

**FIGURE 5:**
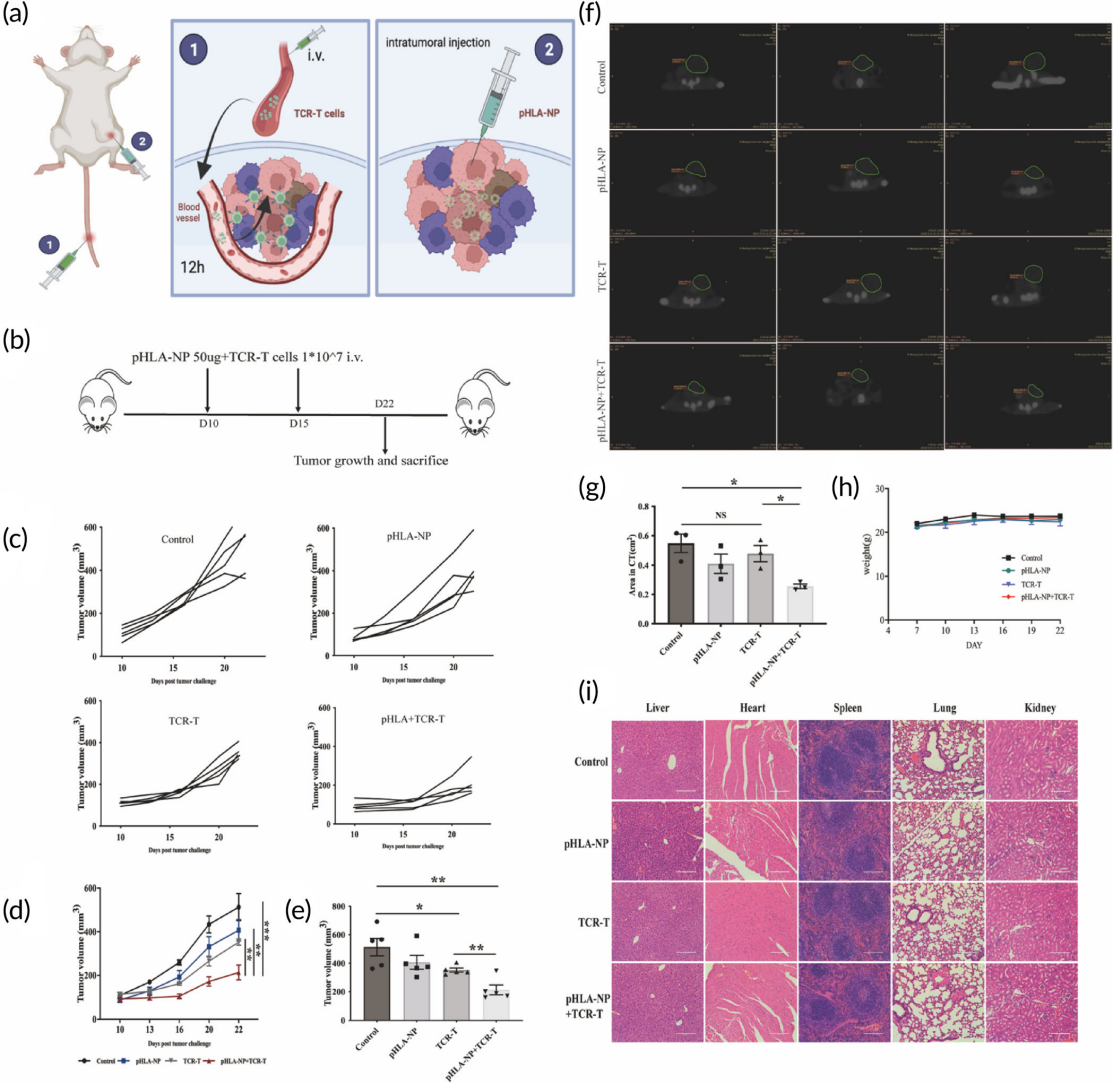
HAUL TCR‐T inhibited the progression of the human GC subcutaneous transplantation model. The nude mice were subcutaneously implanted with 3 × 10^6^ ffLuc+MKN45 cells. The mice were divided into four groups randomly (control group, pHLA‐NP group, TCR‐T group, and pHLA‐NP + TCR‐T group). (a) NY‐ESO‐1‐specific TCR‐T cells were injected into the tail vein, and pHLA‐NPs were injected intratumorally after 12 h. (b) Treatment was performed on the 10th and 15th days after the tumor was implanted. (c, d) Tumor growth profiles. (e) Tumor volume, (f) CT images of each group, and (g) tumor area quantification on CT images on Day 22 after tumor inoculation. (h) The weights of the mice were recorded every 3 days. (i) Organs in all groups were harvested and stained with H&E on Day 22 post tumor implantation. Tumor volumes were analyzed with Student's *t*‐test. Data were represented as Mean ± SEM *n* = 5. Tumor area quantification on CT images (area in CT) was analyzed with Student's *t*‐test. Data were represented as Mean ± SEM *n* = 3. (*p < 0.05; **p < 0.01; ***p < 0.001; ^NS^ *p* > 0.05, not significant).

We next evaluated the anti‐tumor efficacy of delivered TCR‐T cells combined with intratumoral injection of pHLA‐NP. NY‐ESO‐1‐specific TCR‐T cells were injected into the tail vein. pHLA‐NPs were injected intratumorally after 12 h (Figure 5a). Treatment was performed on the 10th and 15th days after the tumor volume reached about 100 mm^3^ (Figure 5b). The tumors in the control group grew rapidly, and the volume in the 22nd day increased by 4.76 times compared with the initial treatment. HAUL TCR‐T could significantly inhibit tumor growth. The final average tumor volume was reduced by approximately 58.43% compared with the control group (Figure 5c–e). CT (Figure 5f,g) and bioluminescence (Figure S6a,b) showed that the HAUL TCR‐T group had a significant anti‐tumor effect compared to the other groups. This experiment showed that the tumor cells were modified by pHLA‐NP, specifically killed by TCR‐T cells in the subcutaneous tumor models. None of the experimental groups exhibited alterations in body weight or vital organ histological morphology (Figure 5h,i), demonstrating the safety of TCR‐T transfer and intratumoral injection of pHLA‐NP.

### 3.5 HAUL TCR‐T inhibited the progression of the human abdominal disseminated gastric tumors

**FIGURE 6:**
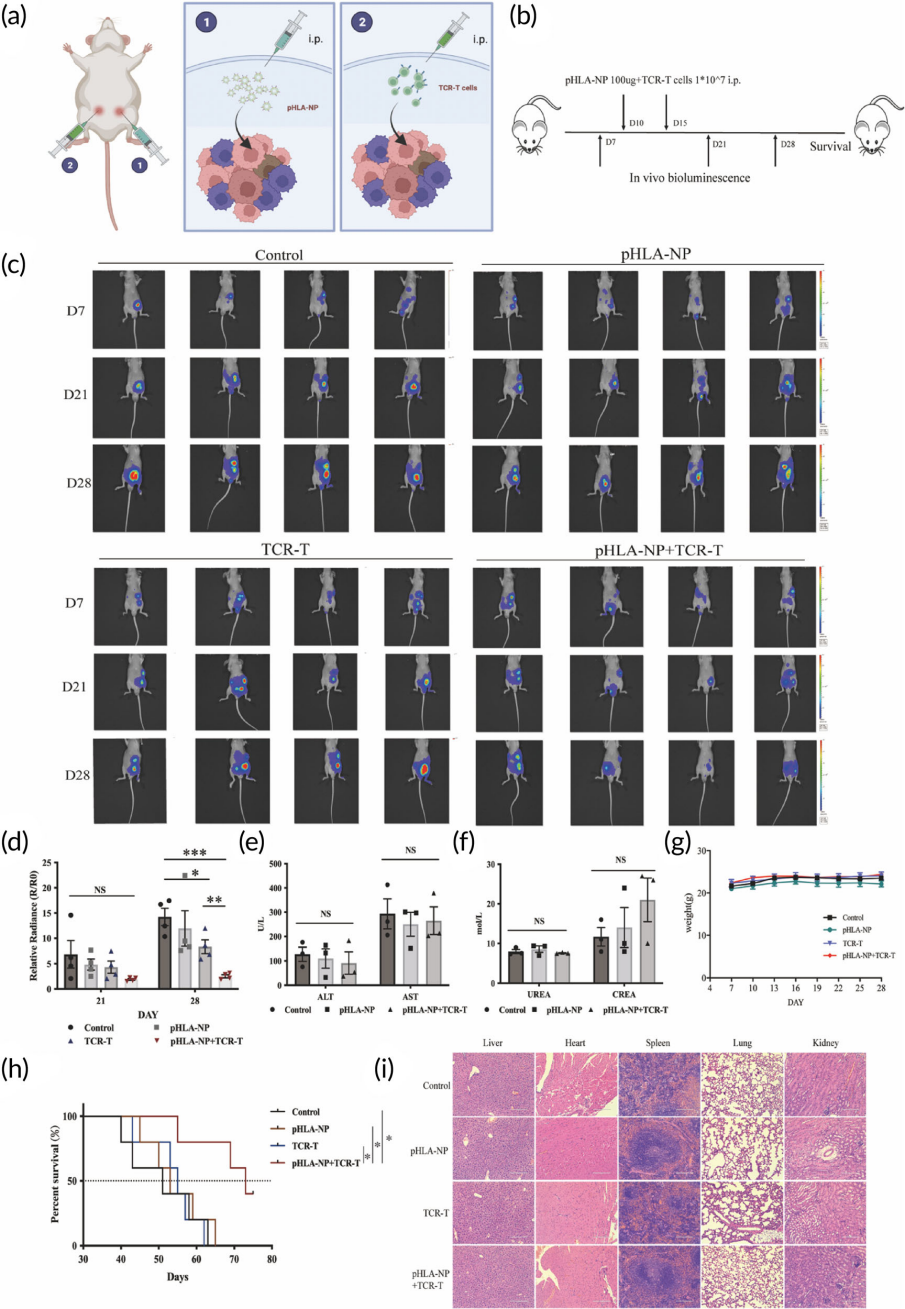
HAUL TCR‐T inhibited the progression of the human GC abdominal disseminated tumor model. The nude mice were subcutaneously implanted with 3 × 10^6^ ffLuc+MKN45 cells. The mice were divided into four groups randomly (control group, pHLA‐NP group, TCR‐T group, and pHLA‐NP + TCR‐T group). (a) pHLA‐NP were injected intraperitoneally into the mice bearing abdominal disseminated gastric tumors. Four hours later, NY‐ESO‐1‐specific TCR‐T cells were injected intraperitoneally. (b) Treatment was performed on the 10th and 15th days after the tumor was implanted. (c) In vivo bioluminescence images of peritoneal metastatic tumor‐bearing mice. TCR‐T and pHLA‐NP were administrated via i.p. infusion. Peritoneal tumor‐killing ability was monitored by changes in bioluminescence on Days 7, 21, and 28. (d) Relative radiances of metastatic peritoneal tumors were calculated on Day 21 and Day 28. Data are represented as Mean ± SEM., *n* = 4. A Student's *t*‐test was used for statistical analysis. (e) Survival curves. Survival curves were analyzed with log‐rank test, *n* = 5. (f) ALT and AST quantification. (g) UREA and CREA quantification. Data are represented as Mean ± SEM, *n* = 3. A Student's *t*‐test was used for statistical analysis. (h) The weights of the mice were recorded every 3 days. Data are represented as Mean ± SEM., *n* = 4. A Student's *t*‐test was used for statistical analysis. (i) Organs in all groups were harvested and stained with H&E on Day 28 post tumor implantation. (*p < 0.05; **p < 0.01; ***p < 0.001; ^NS^ *p* > 0.05, not significant).

We next evaluated the efficacy of this HAUL TCR‐T strategy to treat disseminated abdominal tumors. pHLA‐NP were injected intraperitoneally into the mice bearing abdominal disseminated gastric tumors. Four hours later, NY‐ESO‐1‐specific TCR‐T cells were injected intraperitoneally (Figure 6a). Treatment was performed on the 10th and 15th days after the tumor was implanted (Figure 6b). The fluorescence signal of abdominal tumors in the untreated control group gradually increased over time. It showed that pHLA‐NP alone has no noticeable effect on inhibiting tumor growth. TCR‐T treatment tended to delay the growth of abdominal tumors. HAUL TCR‐T significantly retarded the growth of abdominal tumors, and the relative fluorescence intensity at the observation endpoint was statistically fainter compared with the control group (*p* < 0.001) and the TCR‐T treatment group (*p* < 0.01) (Figure 6c,d).

We also studied the survival period of each group of mice. The median survival time of mice in the control group, pHLA‐NP group, and TCR‐T group were all within 55 days, and the HAUL TCR‐T group could prolong the median survival time of mice to 73 days. On the 65th day, 80% of the mice in the HAUL TCR‐T group still survived, and all the other groups of mice died. On the 75th day of observation, 40% of the mice in the HAUL TCR‐T group were still alive. The above results confirm that HAUL TCR‐T treatment could produce long‐lasting and effective anti‐tumor effects (Figure 6e).

None of the experimental groups exhibited alterations in abnormal liver and kidney function (Figure 6f,g), body weight (Figure 6h), or important organs histological morphology (Figure 6i), which demonstrated that intraperitoneal injection of HAUL TCR‐T was safe.

## 4 DISCUSSION

TCR‐T cells can effectively target tumor antigens presented by HLA molecules, broadening the target spectrum of solid tumor immunotherapy. In 2006, Morgan et al. reported using MART‐1 specific TCR‐T cells to treat melanoma, with an objective response rate (ORR) of 12% (2/17), demonstrating the feasibility of TCR‐T cell therapy in the clinical application.^13^ In 2015, Robbins et al. first showed the clinical effect of TCR‐T cell therapy for NY‐ESO‐1 in patients with melanoma and synovial sarcoma. The ORR of the synovial sarcoma group was 61% (11/18), and the ORR of the melanoma group was 55% (11/20), without toxicity to normal tissue damage.^14^ In 2019, a First‐in‐Human, Phase I/II Study of TCR‐T cell Therapy for HPV‐associated epithelial cancers^15^ showed that HPV16 E6 TCR‐T cells could result in tumor regression and objective responses in patients with chemotherapy‐refractory, metastatic HPV16‐positive epithelial cancer. Neither on‐target autoimmune toxicity nor off‐target cross‐reactivity against healthy tissue appeared. Neoantigen TCR‐T cell therapy for solid tumors, such as pancreatic cancer,^16^ melanoma,^17^ metastatic colorectal cancer,^18^, ^19^ glioblastoma,^20^ metastatic HPV16‐positive cervical, anal, oropharyngeal, and vaginal cancer,^15^ has case reports with good results.

In the treatment of solid tumors, although TCR‐T cell therapy has shown specific anti‐tumor effects in basic research and clinical trials, it still faces some problems before the clinical application of TCR‐T, especially the lack of tumor‐specific targets.^21^ Target problems manifested explicitly in three aspects: (1) HLA‐I down‐regulation results in the lack of T cell recognition targets on the surface of tumor cells^11^, ^12^; (2) Antigen peptide mismatch limits the activation of TCR‐T cells; (3) TCR‐T cell reactivity is restricted to tumor antigen presented by HLA molecules, limiting the type of patient. Current TCR therapies rely on HLA matching.

At present, there are several ways that have been tried to solve the target problems, like injecting specific peptides into tumors^4^ or using carriers like high‐molecular polymer nanospheres to deliver antigens to tumors for specific release.^5^, ^6^ However, there are certain limitations in the way of delivering antigenic peptides. On one hand, the patient's HLA typing must match the antigenic peptide. Otherwise, efficient delivery of the antigen cannot be achieved, significantly reducing this antigenic modification's generality. On the other hand, in the case of tumor HLA down‐regulation or deletion, antigen peptides cannot be effectively presented to the surface of tumor cells, and the modification efficiency of this method will be significantly reduced. Therefore, we imagined whether pHLA molecules could be loaded on the tumor cell membrane, which could overcome the limitation of HLA typing and not be affected by the amount of HLA expression. We used the HLA‐A2‐restricted NY‐ESO‐1 antigen peptide (NY‐ESO‐1_157‐165_) (SLLMWITQC) as the model antigen, which in the TCR‐T clinical trials showed high ORRs in patients and without toxicity to normal tissues.^14^, ^22^, ^23^

The collaborative targeting system can load artificial targets on tumor tissues to support subsequent targeted therapy. The system first delivers the surface‐modified target microspheres to the tumor site as a molecular target. It then injects drugs that can bind to the target molecules on the microspheres so that the medicines can specifically target the tumor site and play a tumor‐killing effect.^8^ Ji‐Ho Park et al. reported that optimized and improved MFL was tumor‐specific and widely labeled in tumor tissues.^7^ A collaborative targeting system using MFL as delivery media was a potent method for loading TCR‐T targets. Based on MFL, we optimized the composition ratio and made it into nanoparticles, namely membrane fusogenic nanoparticles. Cancer cells secrete many lactate anions across plasma membranes as a hallmark. The study of Bingdi Chen et al. revealed that this hallmark may have led to a generation of unique negative charges on the surfaces of cancer cells. Without elevated glycolysis, the surfaces of normal cells remain charge‐neutral or slightly positive.^24^ Therefore, tumor cells may better interact with NPs containing the cationic lipid DOTAP and increase the likelihood of fusion.

In addition, it reported that antigen‐specific Treg cells in mice were activated and expanded after systemic administration of surface‐modified major histocompatibility complex‐peptide (pMHC) microspheres, thereby reducing the symptoms of autoimmune diseases.^25^, ^26^ This result indicated that the artificially prepared pMHC in vitro could be used as the recognition target of antigen‐specific T cells in vivo.

Therefore, we tried to solve the problem of the lack of suitable targets for TCR‐T cell therapy by loading artificial pHLA onto the surface of tumor cells with NPs. To obtain a stable pMHC complex monomer, simplify the production process, and improve the folding efficiency, Yu et al. established the single‐chain trimer (SCT) technology in 2002.^27^ The peptide was always in a covalently bound state and could be firmly bound in the antigen groove. It was easy to recombine even after dissociation, significantly increasing the steady‐state peptide occupancy level.^28^, ^29^ In this experiment, we designed the SCT of pHLA, pHLA was successfully obtained by dilution and renaturation, and improved NPs were prepared. pHLA‐NP was successfully prepared. Confocal results showed that pHLA‐NP could target tumor cells and transfer pHLA onto tumor cell membranes. It was the first attempt to modify pHLA onto tumor cell membranes, adding a specific target for TCR‐T cell therapy.

The in vitro and in vivo experiments showed that pHLA‐NP could modify pHLA on the tumor cell membrane and be specifically killed by TCR‐T cells to improve the anti‐tumor effect. Due to genetic instability, some tumor cells can be immunoedited under selective pressure to reduce the endogenous presentation of antigens. Some tumors have immune escape phenomena such as HLA down‐regulation and loss of neoantigens,^30^, ^31^, ^32^, ^33^ resulting in a lack of T cell recognition targets on the surface of tumor cells. In the in vitro activation and killing experiments, it was still observed that pHLA‐NP modified tumor cells could be specifically recognized and killed by TCR‐T cells. This HAUL TCR‐T cell therapy technology enabled tumor cells to be identified and killed by specific TCR‐T cells, regardless of the HLA typing of tumor cells.

The subcutaneous and abdominal disseminated tumor models had no significant pathological changes in the important organs and weight changes in mice. None of the experimental groups exhibited abnormal liver and kidney function alterations in the abdominal disseminated tumor model, which confirmed the safety of pHLA‐NP and TCR‐T cell therapy in vivo.

We presented for the first time the addition of pHLA on the tumor cell membrane as the target for TCR‐T cell‐specific recognition without HLA restriction, which solved the problem of the lack of effective targets for TCR‐T cell therapy. This article proposed a proof of principle for the HAUL TCR‐T cell therapy, which had translational potential in clinical application for solid tumors.

## AUTHOR CONTRIBUTIONS

**Ruihan Xu:** Methodology (equal); project administration (equal); writing – original draft (equal). **Qin Wang:** Methodology (equal); project administration (equal); writing – original draft (equal). **Junmeng Zhu:** Methodology (equal); writing – review and editing (equal). **Yuncheng Bei:** Methodology (supporting); writing – review and editing (equal). **Yanhong Chu:** Methodology (supporting); writing – review and editing (supporting). **Zhichen Sun:** Methodology (supporting); writing – review and editing (supporting). **Shiyao Du:** Methodology (supporting); writing – review and editing (supporting). **Shujuan Zhou:** Methodology (supporting); writing – review and editing (supporting). **Naiqing Ding:** Funding acquisition (supporting); methodology (supporting). **Fanyan Meng:** Conceptualization (equal); data curation (equal); funding acquisition (equal). **Baorui Liu:** Conceptualization (lead); data curation (equal); funding acquisition (equal).

## FUNDING INFORMATION

This work was funded in part by grants from the National Natural Science Foundation of China (No. 81930080 to Baorui Liu; No. 82072926 to Fanyan Meng; No. 81903128 to Naiqing Ding), Natural Science Foundation of Jiangsu Province (grant number BK20191114 to Fanyan Meng), and Nanjing Medical Science and Technique Development Foundation (No. QRX17038 to Fanyan Meng).

## CONFLICT OF INTEREST STATEMENT

The authors declare no conflicts of interest.

### PEER REVIEW

The peer review history for this article is available at https://www.webofscience.com/api/gateway/wos/peer-review/10.1002/btm2.10585.

## Supporting information


**Figure S1:** The production of pHLA and pHLA‐NP. Recombinant pHLA class I was produced by expressing glycine‐serine (GS) linker‐tethered single‐chain pHLA class I complexes in bacteria. (a) Prokaryotic plasmid map of pHLA. (b) SDS‐PAGE analysis of pHLA expression. Lane 1, protein ladder; Lane 2, bacteria induced with 1 mM IPTG; Lane 3, the supernatant of the ultrasonic lysate; Lane 4, the precipitation of the ultrasonic lysate; Lane 5, solubilized inclusion bodies; Lane 6, Refolded products. (c) Purification via Nickel column after dilution refolding. (d) SDS‐PAGE of Purified products. (e) Schematic illustration of NPs structure. (f) Analysis of the dosage‐effect relationship of NPs mediated modification on tumor cells. (g) Schematic illustration of pHLA‐NP structure. (h) Analysis of the dosage‐effect relationship of pHLA‐NPs mediated modification on tumor cells.
**Figure S2:** pHLA‐NPs did not affect the viability of tumor cells. (a) The state of NUGC4 incubated with pHLA‐NP for 1 h under the microscope. (b) Apoptosis detection was conducted on NUGC4 after pHLA incubation by Annexin V‐FITC/PI. (c) The percentage of Annexin V+ NUGC4 (including early apoptosis and late apoptosis). Data are represented as mean ± s.e.m., *n* = 4. A Student's *t*‐test was used for statistical analysis. ^NS^ *p* > 0.05, not significant.
**Figure S3:** pHLA‐NP could transfer the pHLA onto the surface of tumor cells. NUGC4 was incubated at 37°C, 5% CO_2_ for 1 h, and added with pHLA‐NP or NP. We used His‐tag to locate the pHLA monomer, Dil to mark the cell membrane, and DAPI to stain the nucleus. Confocal images indicated that pHLA was co‐localized with the cell membrane dye DiI.
**Figure S4:** The proportion of NY‐ESO‐1 TCR‐T constructed by tetramer detection on Day 5 and Day 10. The proportion of T lymphocytes with CD8 + tetramer + was about 5.65% on the 5th day after the activated T cells were electroporated. The positive TCR‐T cells were maintained at about 5.03% on the 10th day after the electroporation. The proportion of TCR‐T cells used in the experiment was between 5% and 7%.
**Figure S5:** Killing rates of tumor cells were associated with the dose of nanoparticles. ffLuc+MKN45 cells were co‐cultured with different doses of pHLA‐NP (100, 250, 500 μg/mL) for 1 h as target cells. TCR‐T cells and target cells were co‐cultured at E: T of 5:1, 10:1, 20:1, and 40:1, respectively. Luciferase substrate was added 12 h after incubation and the percentage of dead tumor cells was analyzed according to the values measured by the ultraviolet spectrophotometer.
**Figure S6:** HAUL TCR‐T inhibited the progression of the human GC subcutaneous transplantation model. The 24 nude mice were subcutaneously implanted with 3 × 10^6^ ffLuc+MKN45 cells. The mice were divided into four groups randomly: NS group (*n* = 5), pHLA‐NP group (*n* = 6), NY‐ESO‐1 TCR‐T group (*n* = 6), and pHLA‐NP + NY‐ESO‐1 TCR‐T group (HAUL TCR‐T group) (*n* = 7). (a) Bioluminescence images, (b) tumor signal quantification on Day 22 after tumor inoculation. Data are represented as Mean ± SEM. A Student's *t*‐test was used for statistical analysis. *p < 0.05, ^NS^ *p* > 0.05, not significant.

## Data Availability

The main data supporting the results in this study are available within the paper and its supporting information. Additional data are available on reasonable request.
