# Supplementary material for: Membrane fusogenic nanoparticle‐based HLA‐peptide‐addressing universal T cell receptor‐engineered T (HAUL TCR‐T) cell therapy in solid tumor
Source: Bioeng Transl Med. 2023 Aug 7;8(6):e10585. doi: 10.1002/btm2.10585 (PMC10658479; doi:10.1002/btm2.10585)
Supplement: Supplementary file 1 — Figure S1: The production of pHLA and pHLA‐NP. Recombinant pHLA class I was produced by expressing glycine‐serine (GS) linker‐tethered single‐chain pHLA class I complexes in bacteria. (a) Prokaryotic plasmid map of pHLA. (b) SDS‐PAGE analysis of pHLA expression. Lane 1, protein ladder; Lane 2, bacteria induced with 1 mM IPTG; Lane 3, the supernatant of the ultrasonic lysate; Lane 4, the precipitation of the ultrasonic lysate; Lane 5, solubilized inclusion bodies; Lane 6, Refolded products. (c) Purification via Nickel column after dilution refolding. (d) SDS‐PAGE of Purified products. (e) Schematic illustration of NPs structure. (f) Analysis of the dosage‐effect relationship of NPs mediated modification on tumor cells. (g) Schematic illustration of pHLA‐NP structure. (h) Analysis of the dosage‐effect relationship of pHLA‐NPs mediated modification on tumor cells. Figure S2: pHLA‐NPs did not affect the viability of tumor cells. (a) The state of NUGC4 incubated with pHLA‐NP for 1 h under the microscope. (b) Apoptosis detection was conducted on NUGC4 after pHLA incubation by Annexin V‐FITC/PI. (c) The percentage of Annexin V+ NUGC4 (including early apoptosis and late apoptosis). Data are represented as mean ± s.e.m., n = 4. A Student's t‐test was used for statistical analysis. NS p > 0.05, not significant. Figure S3: pHLA‐NP could transfer the pHLA onto the surface of tumor cells. NUGC4 was incubated at 37°C, 5% CO2 for 1 h, and added with pHLA‐NP or NP. We used His‐tag to locate the pHLA monomer, Dil to mark the cell membrane, and DAPI to stain the nucleus. Confocal images indicated that pHLA was co‐localized with the cell membrane dye DiI. Figure S4: The proportion of NY‐ESO‐1 TCR‐T constructed by tetramer detection on Day 5 and Day 10. The proportion of T lymphocytes with CD8 + tetramer + was about 5.65% on the 5th day after the activated T cells were electroporated. The positive TCR‐T cells were maintained at about 5.03% on the 10th day after the electroporation. The [file BTM2-8-e10585-s001.docx]

*Supporting Information for*

**Membrane fusogenic nanoparticle-based HLA-peptide-addressing universal T cell receptor-engineered T (HAUL TCR-T) cell therapy in solid tumor**

Ruihan Xu^1^, Qin Wang^1^, Junmeng Zhu^1^, Yuncheng Bei^1^, Yanhong Chu^1^, Zhichen Sun^1^, Shiyao Du^1^, Shujuan Zhou^1^, Naiqing Ding^1^, Fanyan Meng^1^, Baorui Liu^1^

**Affiliations:**

^1^ The Comprehensive Cancer Centre of Nanjing Drum Tower Hospital, The Affiliated Hospital of Nanjing University Medical School, Nanjing 210008, China

RX and QW contributed equally.

Correspondence to: Dr Baorui Liu, baoruiliu@nju.edu.cn

Professor Fanyan Meng, fanyanmeng@hotmail.com


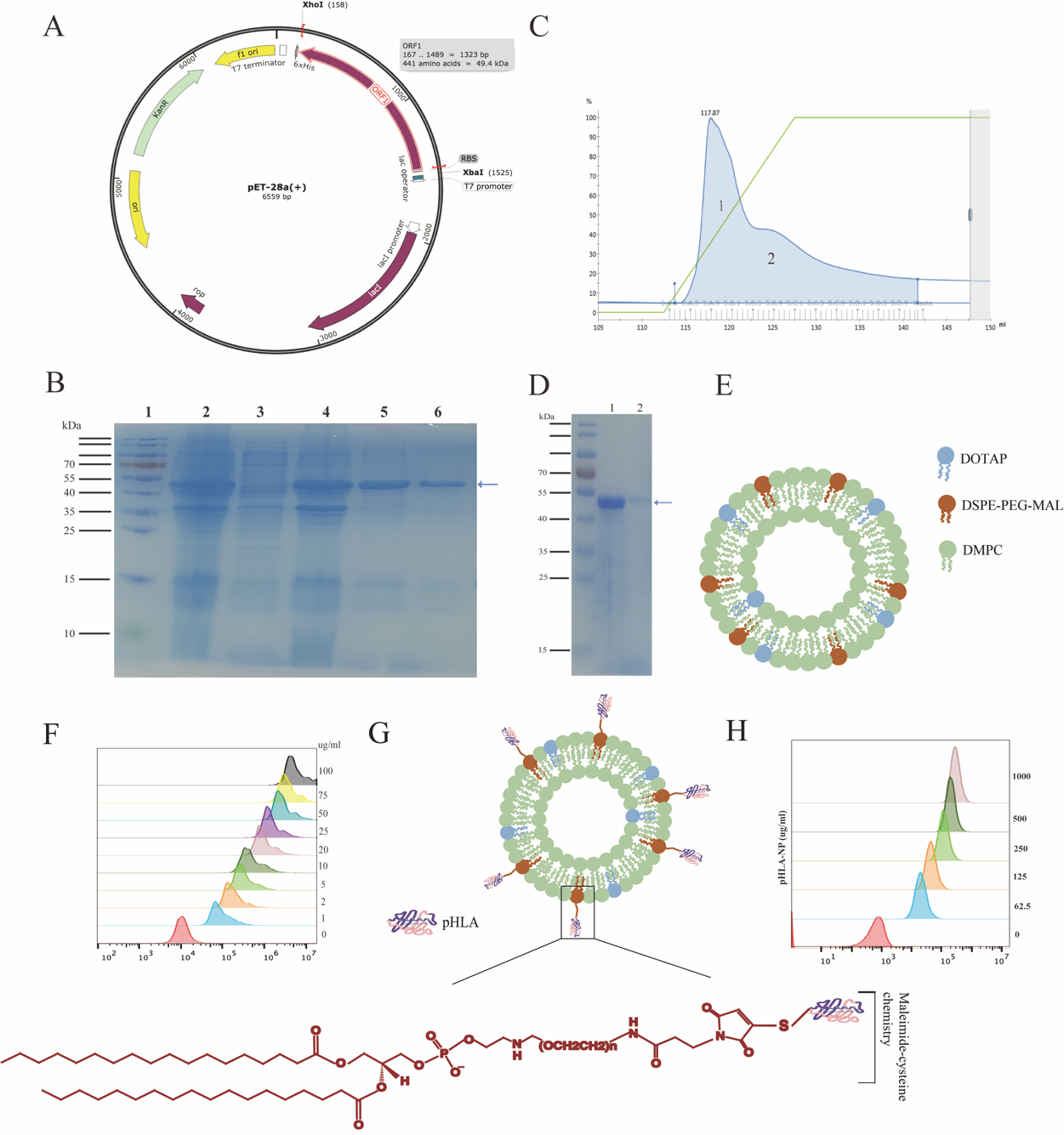


**Figure S1.** **The production of pHLA and pHLA-NP.** Recombinant pHLA class I was produced by expressing glycine-serine (GS) linker-tethered single-chain pHLA class I complexes in bacteria. (A) Prokaryotic plasmid map of pHLA. (B) SDS-PAGE analysis of pHLA expression. Lane 1, protein ladder; Lane 2, bacteria induced with 1 mM IPTG; Lane 3, the supernatant of the ultrasonic lysate; Lane 4, the precipitation of the ultrasonic lysate; Lane 5, solubilized inclusion bodies; Lane 6, Refolded products. (C) Purification via Nickel column after dilution refolding. (D) SDS-PAGE of Purified products. (E) Schematic illustration of NPs structure. (F) Analysis of the dosage-effect relationship of NPs mediated modification on tumor cells. (G) Schematic illustration of pHLA-NP structure. (H) Analysis of the dosage-effect relationship of pHLA-NPs mediated modification on tumor cells.


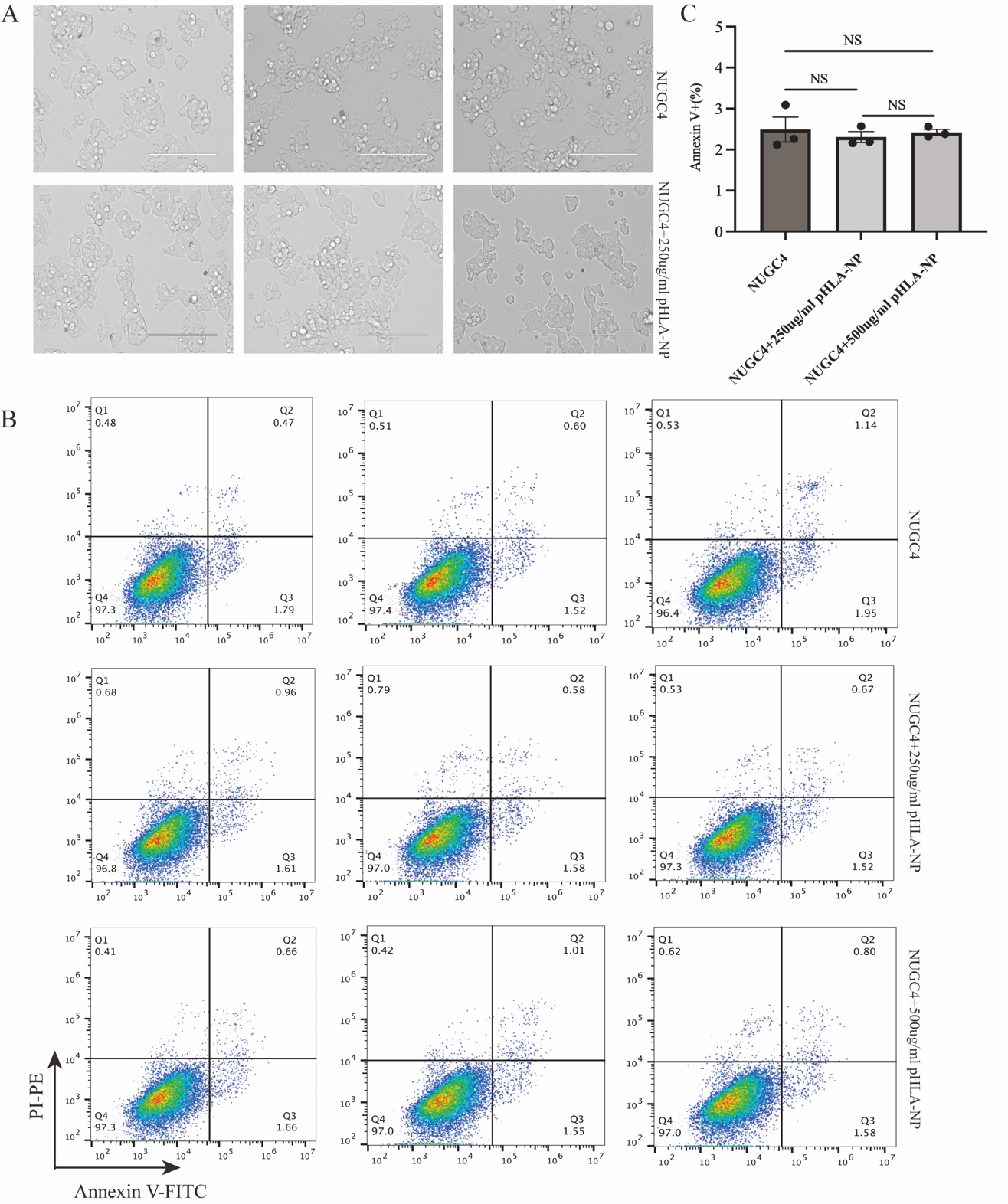


**Figure S2.** **pHLA-NPs did not affect the viability of tumor cells.** (A) The state of NUGC4 incubated with pHLA-NP for 1h under the microscope. (B) Apoptosis detection was conducted on NUGC4 after pHLA incubation by Annexin V-FITC/PI. (C) The percentage of Annexin V+ NUGC4 (including early apoptosis and late apoptosis). Data are represented as mean ± s.e.m., n = 4. A Student’s t-test was used for statistical analysis. ^NS^ P > 0.05, not significant.


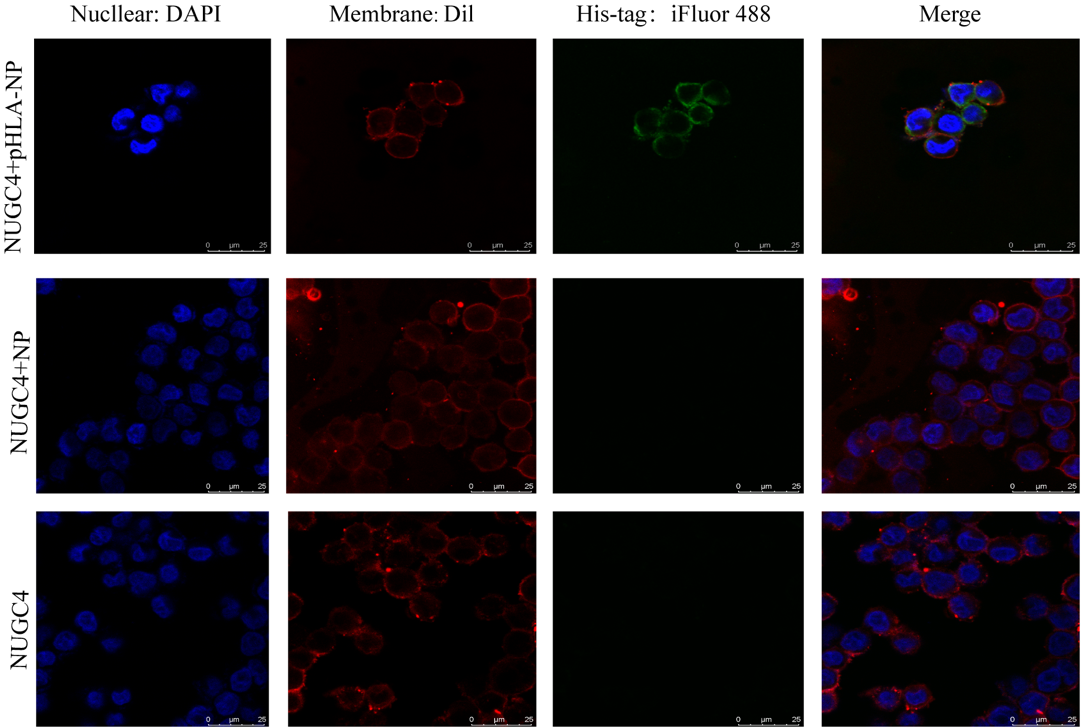


**Figure S3.** **pHLA-NP could transfer the pHLA onto the surface of tumor cells.** Take NUGC4 was incubated at 37°C, 5% CO2 for 1h, and added with pHLA-NP or NP. We used His-tag to locate the pHLA monomer, Dil to mark the cell membrane, and DAPI to stain the nucleus. Confocal images indicated that pHLA was co-localized with the cell membrane dye DiI.

**
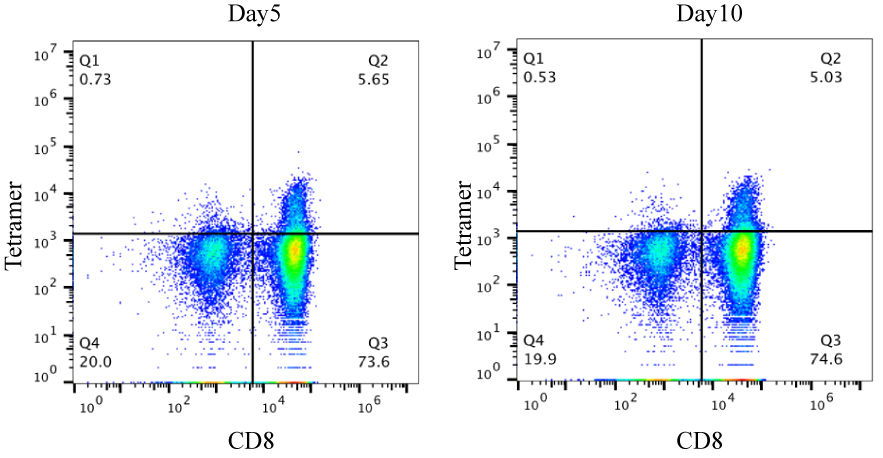
**

**Figure S4.** The proportion of NY-ESO-1 TCR-T constructed by tetramer detection on day5 and day 10. The proportion of T lymphocytes with CD8 + tetramer + was about 5.65% on the 5th day after the activated T cells were electroporated. The positive TCR-T cells were maintained at about 5.03% on the 10th day after the electroporation. The proportion of TCR-T cells used in the experiment was between 5% and 7%.


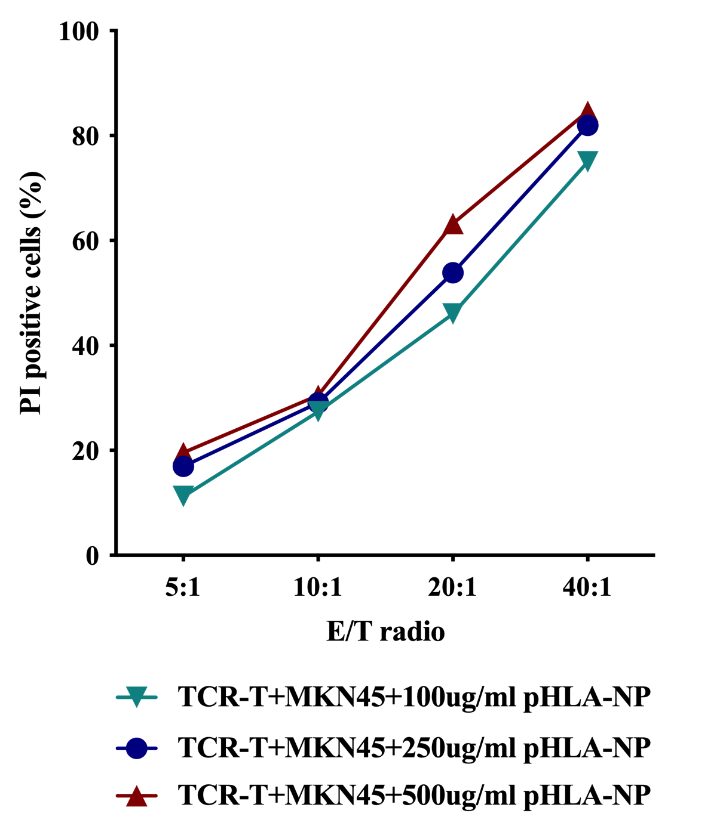


**Figure S5. Killing rates of tumor cells were associated with the dose of nanoparticles.** ffLuc+MKN45 cells were co-cultured with different doses of pHLA-NP (100ug/ml, 250ug/ml, 500ug/ml) for 1 hour as target cells. TCR-T cells and target cells were co-cultured at E: T of 5:1, 10:1, 20:1, and 40:1 respectively. Luciferase substrate was added 12h after incubation and the percentage of dead tumor cells was analyzed according to the values measured by the ultraviolet spectrophotometer.


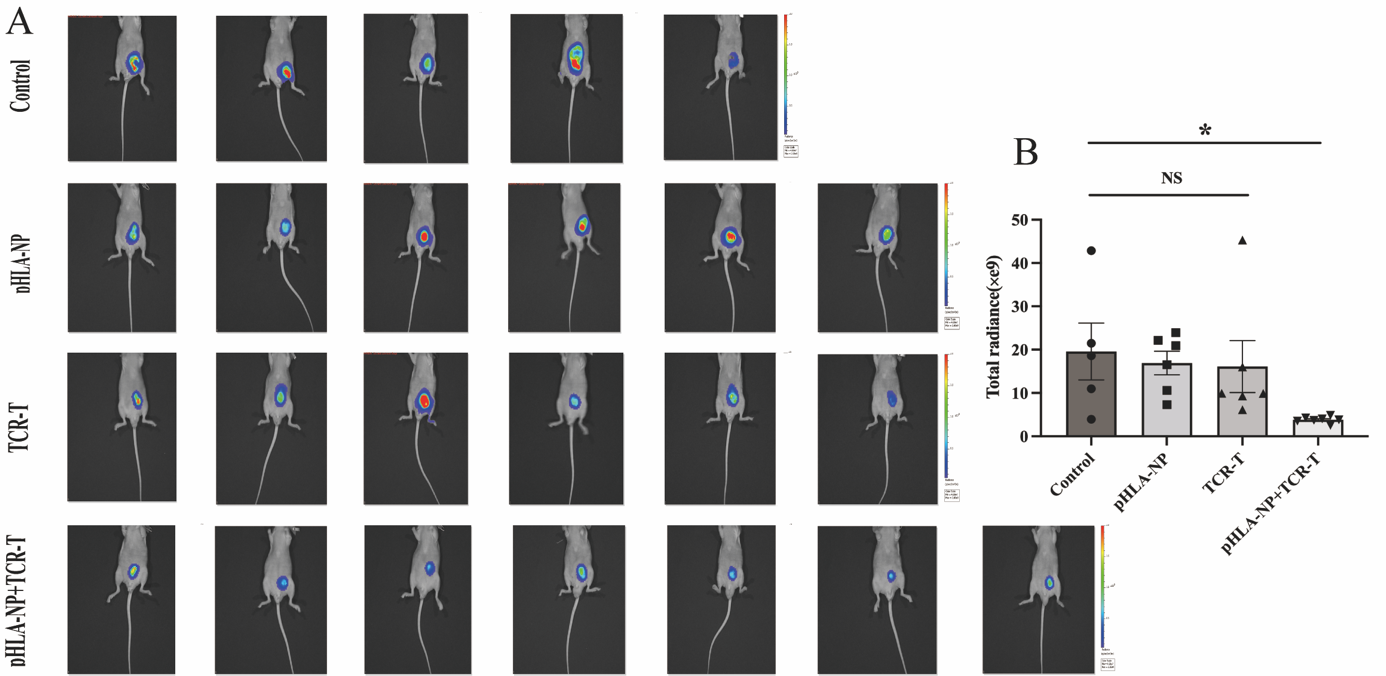


**Figure S6. HAUL TCR-T inhibited the progression of the human GC subcutaneous transplantation model.** The 24 nude mice were subcutaneously implanted with 3 × 10^6^ ffLuc+MKN45 cells. The mice were divided into four groups randomly: NS group (n=5), pHLA-NP group (n=6), NY-ESO-1 TCR-T group (n=6), and pHLA-NP + NY-ESO-1 TCR-T group (HAUL TCR-T group) (n=7). (A) bioluminescence images, (B) tumor signal quantification on day 22 after tumor inoculation. Data are represented as Mean ± SEM. A Student’s t-test was used for statistical analysis. *P < 0.05, ^NS^ P > 0.05, not significant.
